# Bœnninghausen’s Experience with High Potencies

**Published:** 1882-12

**Authors:** 


### BŒNNINGHAUSEN'S EXPERIENCE WITH HIGH POTENCIES.

Bœnninghausen began to practice Homœopathy according to the practical rules laid down by Hahnemann. When the high potencies were first introduced he, at the instigation of Gross, began very cautiously to make experiments with them—first upon domestic animals and afterward, when encouraged by the results, very cautiously upon his patients. Seven years were devoted to these experiments, the results of which were always recorded and carefully collated. Finally, he became convinced of the superiority of the higher over the lower potencies, and for twenty-two years (up to the time of his death) he used only the high potencies; at last, exclusively the 200th in all cases. It was his custom to record every case for which he prescribed. In 1862 he informed the writer that he had just begun the one hundred and twelfth volume of his *Clinical Record*. Of these one hundred and twelve volumes it is safe to estimate that at least eighty contain records of cases treated almost exclusively with high potencies. A rich mine of experience for the conscientious and intelligent explorer!—CARROLL DUNHAM.

One such experience as that of Bœnninghausen's is more valuable than myriads of "*I don't believes.*"

Beside the experience of such a man how puerile and nonsensical does such twaddle as the following read:

As a chief hindrance to the general and candid consideration of the truths of Homœopathy is the absurd doctrine, never taught by Hahnemann*—of infinite dilution, we should endeavor to adopt some standard or limit for drug attenuation, and refuse longer to assume any responsibility for triturations and dilutions made in defiance of all reason and to suit the caprices of men who are satisfied only when surrounded by impenetrable clouds of mysticism. There can be no reasonable objection urged against such action on the part of the Institute. When we remember that ninty-nine out of every hundred homœopathic practitioners rely upon triturations and dilutions within the range ending at the tenth centesimal, and that the great clinical conquests of Homœopathy have been made and nearly all the favorable legislation secured by them [Triturations?—ED.] we are astonished that some such action has not been taken long ago.—BREYFOGLE.

* Dr. Dunham said Hahnemann used "with great success the sixtieth, the one hundred and fiftieth, and the three hundredth dilutions." Dr. Dunham also adds: "It is not unworthy of remark that as Hahnemann's practical experience in the treatment of disease increased, so did his estimate of the advantage and necessity of using the higher dilutions, in at least many cases likewise increase."

The experience of those who so loudly decry high potencies would be similar to that of Hahnemann and Bœnninghausen did they prescribe with equal diligence and accuracy.

